# Association between circulating adiponectin levels and polycystic ovarian syndrome

**DOI:** 10.1186/1757-2215-7-18

**Published:** 2014-02-07

**Authors:** Saira Saeed Mirza, Kashif Shafique, Abdul Rauf Shaikh, Naveed Ali Khan, Masood Anwar Qureshi

**Affiliations:** 1 Department of Epidemiology, University of Rotterdam, Rotterdam, The Netherlands; 2 Institute of Health and Wellbeing, University of Glasgow, 1-Lilybank Gardens, G12 8RZ Glasgow, UK; 3 School of Public Health, Dow University of Health Sciences, Karachi, Pakistan; 4 Department of Community Medicine, Dow University of Health Sciences, Karachi, Pakistan; 5 Department of Surgery, Dow University of Health Sciences, Karachi, Pakistan; 6 Institute of Basic Medical Sciences, Dow University of Health Sciences, Karachi, Pakistan

## Abstract

**Background:**

Low adiponectin levels in polycystic ovarian syndrome (PCOS) have been largely attributed to obesity which is common among these patients. In addition, evidence also suggests that low adiponectin in PCOS may be related to insulin resistance (IR) in these women. However, studies on the role of adiponectin in younger and lean patients are limited. Therefore, the aim of the present study was to examine the association of adiponectin levels in young and lean women with PCOS.

**Methods:**

A case–control study was conducted at the Dow University of Health Sciences, Karachi, Pakistan. Cases were 75 patients of PCOS with Body Mass Index (BMI) &23 aged 16–35 years and 75 healthy age and BMI matched controls were selected from family and friends of the cases. Demographic details, family history and past medical history were obtained through interview by a physician. Anthropometric measurements included weight and height of the participants. Fasting glucose, total cholesterol, high-density lipoprotein (HDL), insulin, adiponectin, and androgen levels were determined. IR was calculated using homeostasis model assessment for insulin resistance (HOMA-IR). Logistic regression models were used to assess the association between adiponectin and PCOS after adjusting for co-variates.

**Results:**

On multivariable analysis, PCOS cases were 3.2 times more likely to have low adiponectin level (OR = 3.2, 95% CI 1.49-6.90, p-value 0.003) compared to the controls after adjustment for age, BMI, family history, marital status, total cholesterol, HDL level and IR. Females with a family history of PCOS were significantly more likely to have lower adiponectin (OR = 3.32, 95% CI 1.27-8.67, p-value 0.014) compared to those who did not have a family history of PCOS. The associations of IR and family history with low adiponectin level also remained statistically significant after adjustments for covariates.

**Conclusion:**

Serum adiponectin levels are independently associated with PCOS and are only partly explained by IR. Adiponectin level may serve as a potential independent biomarker for diagnosis of PCOS in young and lean women with fewer symptoms, or women with a family history of PCOS.

## Introduction

The polycystic ovarian syndrome (PCOS) is the most common endocrine disorder affecting reproductive age women worldwide [1]. Clinical features of PCOS namely hirsutism, acne, and alopecia originate from high circulating levels of androgens, menstrual irregularities from anovulatory cycles [2], and obesity is thought to originate from both the underlying IR [1] and high androgen levels in these patients [3]. The alarming tribulations associated with the syndrome are past the reproductive axis and these women are at a greater risk of developing the metabolic syndrome at an early age because of IR and obesity [4-6] observed in 30-60% of PCOS patients [7].

In recent years, role of adipose tissue hormones, particularly adiponectin has been implicated in the pathogenesis of PCOS [8,9]. Adiponectin has antiatherogenic, anti-diabetic, anti-inflammatory and insulin sensitizing effects, and is negatively related to the degree of adiposity in healthy individuals [10]. Despite being an adipokine, low levels of adiponectin have been found more closely related to the degree of IR than adiposity itself [11]. Studies have also shown that both insulin action and circulating levels of adiponectin are lower in women with PCOS [12].

There are some variations in age at presentation of PCOS between different countries. Furthermore, its typical features like a combination of obesity, hirsutism, acne, alopecia, and irregular menstruation [13] are absent in many women whereas patients manifest with PCOS at a younger age, and without any significant history of its symptoms. Also, obesity and symptoms of hyperandrogenism are also lower in those patients who are lean and presenting a younger age [13].

Although low adiponectin levels have been associated with PCOS which is mainly attributed to obesity among these patients, studies have also suggested that low adiponectin in PCOS may be related to IR in these women. However, the role of adiponectin in younger and lean patients has been examined only in few studies. In these women, it is not certain that to what extent, the IR determines the levels of adiponectin. If adiponectin levels in younger and lean women provide similar association with PCOS as in obese patients, the level of adiponectin may be a useful proxy measure of an ongoing ovarian disease in women with atypical presentation of PCOS. Therefore, we examined the association of adiponectin levels with PCOS in younger and lean women.

## Methods

### Selection of cases

We recruited 75 newly diagnosed PCOS patients aged between 16–35 years with desirable BMI, from the out-patient departments of Gynecology units of two public sector hospitals Civil hospital Karachiand Lady Dufferin hospital, Karachi, Pakistan. Desirable BMI was defined as BMI & 23 according to the Asian reference values for BMI [14]. PCOS was diagnosed using the Rotterdam Criteria [15] which states that PCOS is diagnosed if patient have any two of the following three features, 1) oligo/amenorrhea and/or anovulation, 2) hyperandrogenism and/or hyperandrogenemia, and 3) polycystic ovaries on ultrasound after exclusion of other etiologies. Oligomenorrhea was defined as infrequent menstruation or less than 9 menstrual periods per year. Amenorrhea was defined as absence or abnormal cessation of menses for three months or more [15]. For diagnostic purposes, since we recruited already diagnosed patients of PCOS, presence of either clinical hyperandrogenism or biochemical hyperandrogenemia was considered acceptable, whichever used by the diagnosing gynecologist. Hyperandrogenism was defined as a score of 7 or more on the Ferriman Gallaway index, or apparent severe hirsutism, acne and alopecia [2].

We did not include pregnant PCOS patients. In addition, patients with type 2 diabetes mellitus, chronic liver disease, thyroid dysfunction, and using medications such as steroids, contraceptives, hypoglycemic/antidiabetic drugs were not included in the study.

### Selection of controls

Controls were 75 age-matched healthy females with regular menstrual cycle from family and friends of the cases. Controls also had BMI within desirable range. Females taking medication, including steroids and contraceptives were not included.

Demographic information, detailed menstrual and reproductive history, family history of menstrual or reproductive problems, past medical history, and anthropometric profile were recorded. Fasting blood samples were drawn from all participants for assessment of blood glucose, lipid profile, adiponectin, insulin and androgen levels. Fasting serum adiponectin was estimated using the Bio-Rad PR 3100 which uses Enzyme Linked ImmunoSorbent Assay (ELISA) technique of quantitative hormone estimation. Adiponectin was categorized using the median value of the sample, 13.0 μU/ml.

Written informed consent was obtained from all study participants. The study was approved by the Ethics Committee/Institutional Review Board (IRB), Dow University of Health Sciences.

### Covariates

Age, marital status, family history of PCOS, BMI, IR, total blood cholesterol level and HDL were used as covariates. Age was categorized into 5-year age groups. For marital status, women were grouped into married and unmarried. Family history was inquired during the interview. Participants were asked questions about the presence/history of the following complaints in their first degree (mother and/or sisters), and/or second degree (aunts and/or cousins) relatives:

1. History of PCOS

2. History of any menstrual problems

3. Problems in conception

4. Excess facial hair, baldness, and/or acne resistant to treatment

From the last three questions, presence of any two, was also considered as a positive history of PCOS, and finally participants were categorized as having a negative or a positive family history of PCOS.

BMI was calculated using the standard formula. IR was calculated using HOMA-IR [16]. HOMA-IR calculates the IR by dividing the product of fasting blood glucose level (mg/dl) and serum insulin level (μU/ml) by a constant, i.e. 405. A HOMA-IR value of 2.5 or above were considered as insulin resistant [16]. Fasting blood glucose was estimated using the automatic biochemical analyzer (Hitachi 902) which uses the photometric technique of glucose estimation. Fasting serum insulin was estimated, using IMMULITE 1000 analyzer which is a solid-phase, two site chemiluminescentimmunometric assay. Lipid profile, (total serum cholesterol and HDL were the parameters of interest) was done by enzymatic calorimetric test. We measured free serum testosterone in fasting state; hyperandrogenemia was defined as free serum testosterone levels higher than 100 ng/ml [17].

### Statistical analysis

Data were analyzed using the STATA Software Version 12 (StataCorp, College Station, TX, USA). Results are presented as means and standard deviations. Threshold for statistical significance was set at p & 0.05. Socio-demographic and other biochemical measures were compared between cases and control using the independent sample *t*-test and chi-squared test for continuous and categorical variables, respectively. To analyze the association between study variables, adiponectin was used as the dependent variable and age, marital status, family history of PCOS, BMI, total cholesterol, HDL and IR were used as independent variables in logistic regression models. To assess the association between adiponectin with PCOS, both univariable and multivariable models were used. Multivariable regression model included age, marital status, family history of PCOS, BMI, total cholesterol, HDL, and IR as co-variates.

## Results

A total of 150 individuals participated in this study, of which 75 were diagnosed PCOS cases and 75 controls. The mean age of sample was 25.6 (SD 6.12), with no statistically significant difference between cases and controls (p-value 0.76). The majority of participants were married (n = 80, 53.3%), with no significant difference in distribution of married individuals between cases and controls (p-value 0.33). Furthermore, there was no significant difference in total cholesterol (p-value 0.72), HDL (p-value 0.19) and family history of PCOS among cases and controls (p-value 0.08). However, there were significant differences in BMI and adiponectin level between cases and controls. PCOS Cases had significantly higher BMI (mean difference 0.97, p-value .02) and lower adiponectin level (mean difference 5.73, p-value &0.001). The demographic and other characteristics of study sample are described in Table 1.

**Table 1:** Socio-demographic and biochemical characteristics of PCOS cases and controls

	**Cases**		**Controls**		**P-value**
**Characteristics**	**n**	(%)*	**n**	(%)*	
**Total participants**	75	(50.0)	75	(50.0)	
**Age, mean(SD)**	25.7	(6.0)	25.4	(6.3)	0.76
**Age, categorical**						
16-20	17	(22.7)	19	(25.3)	0.98	
21-25	22	(29.3)	22	(29.3)		
26-30	12	(16.0)	11	(14.7)		
31-35	24	(32.0)	23	(30.7)		
**Marital status**						
Unmarried	38	(50.7)	32	(42.7)	0.33	
Married	37	(49.3)	43	(57.3)		
**Family history of PCOS**						
No	63	(84.0)	54	(72.0)	0.08	
Yes	12	(16.0)	21	(28.0)		
**Body mass index, mean (SD)**	19.3	(2.6)	18.3	(2.4)	0.02	
**Total cholesterol**						
&6.2	68	(90.7)	69	(92.0)	0.72	
≥6.2	7	(9.3)	6	(8.0)		
**High density lipoprotein**						
&1.29	35	(46.7)	43	(57.3)	0.19	
≥1.29	40	(53.3)	32	(42.7)		
**Insulin resistance**	3.1	(2.1)	2.9	(2.7)	0.73	
**Adiponectin level, mean (SD)**	12.4	(2.7)	18.2	(7.8)	&0.01	
**Adiponectin level, categorical**						
≤13.0	27	(36.0)	45	(60.0)	&0.01	
>13.0	48	(64.0)	30	(40.0)		

*n and percentages are mentioned until stated otherwise.

Univariable analysis to assess the association between PCOS and adiponectin level (adiponectin ≤13.0), revealed that cases were 2.7 times more likely (OR = 2.67, 95% CI 1.38-5.16, p-value 0.004) to have lower adiponectin compared to controls. Females with a family history of PCOS were significantly more likely to have to lower adiponectin (OR = 3.11, 95% CI 1.33-7.26, p-value 0.009) compared to those who did not have a family history of PCOS (Table 2). IR also showed a statistically significant negative association with low adiponectin (p-value 0.001). Other factors including age, BMI, marital status, total cholesterol level and HDL did not show statistically significant association with adiponectin (Table 2).

**Table 2:** The relationship of PCOS and other characteristics of low adiponectin level

	**Univariate analysis**			**P-value**	**Multivariate analysis**		**P-value**
**Characteristics**	**Odds ratio**	**(95% CI)**			**Odds ratio**	**(95% CI)**	
**PCOS status**							
Controls	1				1		
Cases	2.67	(1.38-5.16)	0.004	3.20	(1.49-6.90)	0.003	
**Age, categorical**							
16-20	1				1		
21-25	0.54	(0.22-1.32)	0.179	0.53	(0.19-1.43)	0.207	
26-30	1.34	(0.45-3.96)	0.597	0.92	(0.17-4.89)	0.924	
31-35	0.68	0.29-1.64)	0.396	0.57	(0.12-2.71)	0.479	
**Marital status**							
Unmarried	1				1		0.928
Married	0.94	(0.49-1.78)	0.844	1.06	(0.27-4.13)		
**Family history of PCOS**							
No	1				1		0.014
Yes	3.11	(1.33-7.26)	0.009	3.32	(1.27-8.67)		
**Body mass index, mean (SD)**	1.09	(0.96-1.24)	0.163	0.97	(0.84-1.13)	0.721	
**Total cholesterol**							
&6.2	1				1		0.268
≥6.2	1.53	(0.47-4.92)	0.474	2.06	(0.57-7.40)		
**High density lipoprotein**							
&1.29	1				1		0.482
≥1.29	1.63	(0.86-3.12)	0.137	1.30	(0.63-2.69)		
**Insulin resistance**	1.29	(1.11-1.52)	0.001	1.23	(1.03-1.47)	0.023	

Multivariate model include all covariates presented in this table.

On multivariable analysis, the overall findings of univariable analysis remained consistent. PCOS cases were 3.2 times more likely to have low adiponectin level (OR = 3.2, 95% CI 1.49-6.90, p-value 0.003) compared to controls after adjustment for age, BMI, family history, marital status, total cholesterol level, HDL and IR. The associations of IR and family history with low adiponectin level also remained statistically significant after adjustments for covariates (Table 2).

**Figure 1:**
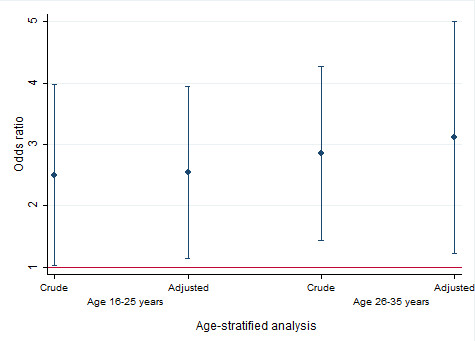
**Age-specific relationship between PCOS and adiponectin level.** Adjusted odds ratio are estimated after accounting for BMI, family history of PCOS, marital status, total cholesterol level, high density lipoprotein level and insulin resistance.

A stratified analysis was also carried out based on age categories (≤25 years and >25 years) to assess the association of low adiponectin with PCOS. PCOS cases of age ≤25 years were 2.5 times more likely to have low adiponectin while cases of age >25 years were 2.9 times more likely to have low adiponectin, compared to their respective control groups (Figure 1). These associations changed slightly and remained statistically significant after adjustment for age, BMI, family history, marital status, total cholesterol level, HDL level and IR (Figure 1).

## Discussion

Our findings suggest that PCOS women with a desirable BMI are significantly more likely to have low serum adiponectin levels. The association of PCOS with low adiponectin level remained consistent and statistically significant after adjustment for age, BMI, family history of PCOS, marital status, total cholesterol level, HDL and IR. This relationship between PCOS and low adiponectin also changed a little across different age groups. Furthermore, family histories of PCOS and IR were also significantly associated with lower adiponectin levels. We found low levels of adiponectin in lean young women with PCOS. Several studies have demonstrated reduced levels of serum adiponectin in women with PCOS [12,18-23]. Also, few of them have shown an association of low adiponectin levels in PCOS women irrespective of the weight and/or BMI of patients [12,18,20-22].

A systematic review and meta-analysis by Toulis et al., on a sub-analysis by using studies only with PCOS cases and controls matched on BMI, revealed that PCOS women had lower levels of adiponectin after controlling for the potential effects of obesity by BMI matching. This suggests that serum adiponectin levels are not independently determined by the degree of adiposity in women but underlying disease may also have some role. A possible explanation for this finding is that lower adiponectin levels in PCOS women might be a result of increasing IR in these patients [1] as supported by our study. As adiponectin is known to possess insulin-sensitizing, anti-diabetic properties and reduced circulating levels are also observed in type 2 diabetes mellitus [24], IR might possibly be a link between lower adiponectin level and development of polycystic ovarian syndrome; however, whether low adiponectin is a cause or a consequence of IR in PCOS remains debated. In addition, it has been observed in randomized controlled trials that treatment of PCOS patients with anti-diabetic medication as metformin [25,26], rosiglitazone [27], and pioglitazone [28], in addition to reductions in secretion of insulin and improvement in its action on glucose metabolism, also increases the adiponectin levels in circulation. However, in multivariable analysis, we observed lower adiponectin levels in women with PCOS, after adjusting for all possible confounders stated, including IR. Stratified analysis (data not shown) based on IR also showed a lower adiponectin level in PCOS women, suggesting that adiponectin levels in these women are regulated by certain unexplained factors other than IR. It could be possible that genetically predisposed women to PCOS might exhibit a lower secretion of adiponectin which may lead to other features/symptoms of PCOS with time.

We have also found an association of family history of PCOS and IR with lower adiponectin levels in PCOS women. Increasing evidence suggests that genetic factors play an important role in the pathogenesis of PCOS. Interestingly, prevalence of PCOS in South East Asians settled in United Kingdom was 52%, which is an 18% higher rate than the native population, suggestive of some genetic predisposition of PCOS among certain races [29]. In addition to familial clustering of PCOS in first degree relatives [30], it has been shown that the pre-pubertal daughters with normal BMI, of women with PCOS, manifest with disturbed metabolic profile including hypoadiponectenemia and hyperinsulinemia compared to daughters of healthy women [31]. In our study, controls were recruited from the family and friends of the cases. Cases and controls did not differ significantly regarding the history of PCOS. This might be argued that as history of PCOS is strongly related to the incidence of PCOS, however, it is not necessary that persons having PCOS always have a history of PCOS. This points towards the hypothesis that, although family history is an important risk factor, environmental triggers are also playing a role, e.g. diet, and exercise. In addition, PCOS is a syndrome and a polygenic causality cannot be ruled out. Dysfunctional changes in the metabolism of carbohydrates, insulin action, and steroid hormones have also been implicated. Therefore, a similar family history of PCOS can be explained as being one of the many contributory factors that cause PCOS, and a similarity of family history in cases and controls is not contradictory.

We found significantly lower levels of adiponectin in PCOS patients &25 years of age. It is possible that the younger age-group is the group with the involvement of a stronger genetic component in PCOS pathogenesis, which lead to an early manifestation of disease. This might indicate a more severe disease phenotype, which may worsen overtime, and become more resistant to treatment. Longitudinal studies can provide further insights, to better understand the course of disease in such patients. As we included lean women with PCOS, which is also a common presentation in clinics in Pakistan, our data suggest that lower adiponectin levels in women with PCOS are not only caused by the IR and obesity in these women. On the contrary, it might be interplay of family history of PCOS and IR, or these women have inherent low levels of adiponectin regardless of their BMI and degree of IR, which leads to development of the full-blown PCOS. Also, a positive family history of PCOS may evoke a disturbance in the insulin secretion in genetically predisposed individuals, giving rise to lower adiponectin levels and polycystic ovaries. Adiponectin thus may serve as a useful marker in detecting cases of PCOS with atypical presentation or in individuals with a family history of PCOS. Longitudinal studies are therefore warranted to understand the initiating point in development of PCOS in females having a family history of PCOS and IR.

### Strengths and limitations

To our knowledge, this is the first study to investigate serum adiponectin levels in PCOS women in Pakistani population. However certain methodological considerations are worth-mentioning. Sample size was limited and the effect estimates may not be very precise, however, we found a statistically significant relationship between PCOS and adiponectin which remained significant even with this smaller sample. In addition, we calculated IR using HOMA-IR and not by the euglycemic/hyperglycemic clamp which is the gold standard to measure IR. However, HOMA-IR is a worldwide accepted surrogate marker for the calculation of IR. This may have potentially misclassified some individuals, however such misclassifications are likely to be non-differential and should lead to a null results. But in our study, we observed a statistically significant association both for IR and adiponectin with PCOS, which is unlikely due to a misclassification bias.

## Conclusion

In conclusion, serum adiponectin levels in lean women with PCOS are only partly explained by IR. Adiponectin levels may serve as a potential independent biomarker for diagnosis of PCOS in lean women with fewer symptoms, or women with a family history of PCOS. Further research using prospective design may provide evidence on role of adiponectin in early diagnosis or detection of PCOS among young lean women.

## Competing interests

All authors declare that they have no competing interests.

## Authors’ contributions

SSM, MAQ designed the study; KS, SSM and NAK carried out statistical analyses; all authors interpreted the results. SSM and NAK drafted the initial manuscript and all authors contributed to the final draft. MAQ supervised the research project. All authors read and approved the final manuscript.

## Funding

Dow University of Health Sciences (DUHS) provided partial funding for biochemical tests of cases of PCOS. DUHS had no role in design, conduct and analysis of this study.
